# Weakly supervised end-to-end artificial intelligence in gastrointestinal endoscopy

**DOI:** 10.1038/s41598-022-08773-1

**Published:** 2022-03-22

**Authors:** Lukas Buendgens, Didem Cifci, Narmin Ghaffari Laleh, Marko van Treeck, Maria T. Koenen, Henning W. Zimmermann, Till Herbold, Thomas Joachim Lux, Alexander Hann, Christian Trautwein, Jakob Nikolas Kather

**Affiliations:** 1 Department of Medicine III, University Hospital RWTH Aachen, Pauwelsstrasse 30, 52074 Aachen, Germany; 2 Department of Medicine, Rhein-Maas-Klinikum, Würselen, Germany; 3 Department of Visceral Surgery and Transplantation, University Hospital RWTH Aachen, Aachen, Germany; 4 Interventional and Experimental Endoscopy (InExEn), Internal Medicine II, University Hospital Würzburg, Würzburg, Germany; 5 Division of Pathology and Data Analytics, Leeds Institute of Medical Research at St James’s, University of Leeds, Leeds, UK; 6 Medical Oncology, National Center for Tumor Diseases (NCT), University Hospital Heidelberg, Heidelberg, Germany

**Keywords:** Colonoscopy, Oesophagogastroscopy, Image processing, Machine learning, Diagnostic markers

## Abstract

Artificial intelligence (AI) is widely used to analyze gastrointestinal (GI) endoscopy image data. AI has led to several clinically approved algorithms for polyp detection, but application of AI beyond this specific task is limited by the high cost of manual annotations. Here, we show that a weakly supervised AI can be trained on data from a clinical routine database to learn visual patterns of GI diseases without any manual labeling or annotation. We trained a deep neural network on a dataset of N = 29,506 gastroscopy and N = 18,942 colonoscopy examinations from a large endoscopy unit serving patients in Germany, the Netherlands and Belgium, using only routine diagnosis data for the 42 most common diseases. Despite a high data heterogeneity, the AI system reached a high performance for diagnosis of multiple diseases, including inflammatory, degenerative, infectious and neoplastic diseases. Specifically, a cross-validated area under the receiver operating curve (AUROC) of above 0.70 was reached for 13 diseases, and an AUROC of above 0.80 was reached for two diseases in the primary data set. In an external validation set including six disease categories, the AI system was able to significantly predict the presence of diverticulosis, candidiasis, colon and rectal cancer with AUROCs above 0.76. Reverse engineering the predictions demonstrated that plausible patterns were learned on the level of images and within images and potential confounders were identified. In summary, our study demonstrates the potential of weakly supervised AI to generate high-performing classifiers and identify clinically relevant visual patterns based on non-annotated routine image data in GI endoscopy and potentially other clinical imaging modalities.

## Introduction

Artificial intelligence (AI) with deep convolutional neural networks has transformed image analysis in medicine. While diagnosing diseases from image data was for decades the unquestioned territory of highly trained domain experts, in 2017, the first application of deep learning in medical routine images changed this conception fundamentally^1^. Clinically relevant tasks are now being solved by AI across multiple data modalities in medicine, including dermatoscopy^2^, histopathology^3^, radiology^4^ and endoscopy^5^. These AI systems are expected to transform patient care and provide improved outcomes for patients and resource savings for the healthcare system in the future^6^. In particular, AI is expected to have a profound effect on diagnosis and treatment of diseases of the gastrointestinal (GI) tract, including cancer of the gut and liver^7,8^, inflammatory diseases^9^ and other diseases of major epidemiological importance.

Endoscopy of the upper and lower GI tract (gastroscopy and colonoscopy) is ubiquitously used to diagnose and treat a wide range diseases. For example, colonoscopy is used to screen for and treat colorectal adenomas, thereby preventing cancer^10^. The broad implementation of colonoscopy-based screening has had a major public health impact and is currently being recommended by international medical guidelines^11^. AI has been successfully used to automate this screening procedure by automatically delineating adenomas in colonoscopy image data^12–16^. These approaches have to date yielded multiple commercially available deep learning-based systems for polyp (adenoma) detection which are successfully being applied in endoscopy routine across the world. A broad body of evidence has demonstrated that these AI systems can improve diagnostic performance while maintaining procedure times^17^. In addition to adenoma detection, AI has been used for other tasks in GI endoscopy, including detection of helicobacter infection^18^, detection of gastric cancer^19^, precancerous lesions of the esophagus^20,21^, automating standardized reporting of endoscopy procedures^22^ and a broad range of other clearly defined disease detection tasks^23^.

Virtually all of these AI approaches use supervised workflows. This means that the pattern of interest is manually delineated in endoscopy images and these manual annotations are used as a ground truth to train an AI system. A less laborious alternative to these region-specific annotations is to manually generate labels for images. However, any form of manual annotations requires involvement of human experts whose time and effort is highly limited and not scalable. Consequently, manual annotations are currently the bottleneck for development and validation of AI systems in endoscopy. Considering that endoscopy is being used to treat a multitude of diseases, supervised training of AI systems on all possible diseases is not feasible. In other words, the conventional AI studies use a bottom-up approach—they aim at detecting pre-defined and manually segmented pathologies^24^ and this approach is not scalable in practice. An alternative is a top-down approach, making use of the vastly diverse imagery obtained by upper and lower gastrointestinal endoscopy and linking these to high-level descriptive data in clinical routine. Such approaches have been successfully used in other fields of medical AI^25,26^ and—although it bears the potential of biases and confounders^27^—can be highly efficient at the same time discover previously unknown or underappreciated visual patterns.

Therefore, in this study, we aimed to develop an AI system for detection of diseases in upper and lower GI endoscopy just by using diagnostic information from routine reports, without using any dedicated manual annotation of images. We deliberately included diagnostic categories related to known endoscopic image patterns (such as “rectal cancer” [C20]) as well as clinical diagnoses present in the endoscopy reports (such as “abdominal and pelvic pain” [R10]). We aimed to estimate the diagnostic performance that can be obtained by such weakly supervised AI systems with respect to endoscopic and clinical diagnoses and investigated the explainability of such systems as well as the potential susceptibility to biases in the dataset.

## Methods

### Ethics statement

This study was performed according to the Declaration of Helsinki. The retrospective analysis of routine endoscopy images and the use of anonymized results for quality improvement and research purposes was approved by the Clinical Advisory Board (CAB) board of University Hospital Aachen on 15th April 2021 and the Ethics Board of the Medical Faculty of RWTH Aachen University on 30th April 2019 (EK 030/19). The Ethics Board of the Medical Faculty of RWTH Aachen University waived the need for informed consent for this retrospective analysis of anonymized data. In addition, the retrospective analysis of routine endoscopy images was approved by the ethics board of the University Hospital Wuerzburg on 28th January 2021 under the project title “Training of artificial intelligence with retrospectively collected anonymized endoscopy images and endoscopy videos for better recognition and classification of pathologies using standardized terminology” (ethics ID 2020011404).

### Endoscopy data set

As a primary dataset for all analyses in this study, we obtained endoscopy reports for all gastroscopy and colonoscopy exams at University Hospital of RWTH Aachen University which were performed between January 2010 and April 2021 (colonoscopy, 23,439 examinations) and between January 2011 and April 2021 (gastroscopy, 39,989 examinations), respectively. We then obtained all endoscopy images which were stored in the routine database Clinic WinData (E&L medical systems, Erlangen, Germany) and matched images to reports by using a unique examination ID. This procedure yielded 18,942 colonoscopy examinations with a total of 164,133 individual images and 29,506 gastroscopy examinations with a total of 209,041 images. No other selection of data was performed. Most raw images had patient-related information (name, date of birth) written on the images, although after cropping, most of this information was removed. All analyses were run on an on-premise computing server within the University Hospital. All images which were exported for visualization were manually anonymized by placing black bars on all patient-related information using Inkscape (https://inkscape.org/). This anonymization was done after the actual analysis, solely for the purposes of creating the figures in this publication. As an additional external validation set, we obtained routine images of endoscopy examinations from the University Hospital Wuerzburg (Wuerzburg, Germany). This validation set comprised 40 gastroscopy examinations (among these, 10 examinations for each of the diagnosis categories B37, C15 and C16) and 40 colonoscopy examinations (among these, 10 examinations for each of the categories C18, C20 and K57). All data were stored in and extracted from the software Clinic WinData, Version 9.7.2 (E&L medical systems GmbH, Erlangen, Germany).

### Image preprocessing

All images were preprocessed with a custom-written script in Matlab R2021a (Mathworks, Natick, USA). To remove black margins around the images, an automatic cropping procedure was performed, which removed these margins and resulted in a square image. Specifically, 25 pixels were cropped at the top and bottom, 40 pixels were cropped at the left side of the image and the image was cropped to a 1:1 ratio, aligned at the left side of the image. The parameters for the cropping were identical for the whole dataset and thus did not introduce a selection bias. Subsequently, adaptive histogram normalization was performed in 64 tiles of the image with a clip limit of 0.005 using the “adapthisteq” function. Images were then resized to 1024 × 1024 px and were stored on the disk. Before the deep learning process, images were resized to 512 × 512 px for transfer learning. On a subset of targets (C18, C20, K50, K51), we trained networks with an input size of 224 × 224 or 1024 × 1024 pixels and did not observe a consistent improvement in performance compared to the baseline model with 512 × 512 px input size.

### Ground truth labels

To create routine reports, endoscopists use predefined macros to generate diagnosis texts. These macros also create an international classification of diseases (ICD) code which is saved as part of the report. This results in a reliable ICD code for every examination set by a physician experienced in gastrointestinal endoscopy directly after the examination. We extracted all ICD10 codes from the reports and selected the most common codes (all codes which occurred more than 60 times), yielding 23 and 19 diagnosis codes in the gastroscopy and colonoscopy data set, respectively (Tables 1 and 2). We then used one-hot encoding to represent these diagnosis codes and used them as a ground truth for all subsequent analysis steps. All ground truth labels were defined on the level of examinations. If multiple ICD codes were present in each examination report, the examination was considered as “positive” for each ICD code and “negative” for all other ICD codes. Examinations had multiple images, all of which inherited the ground truth label of the parent examination.

**Table 1:** Target diseases and classifier performance for the gastroscopy dataset.

ICD	Diagnosis	AUROC mean	AUROC 95% CI	N pos exams	N neg. exams	p-val
K29	Gastritis and duodenitis	0.698	0.007	9740	19,766	0.0000
K44	Diaphragmatic hernia	0.637	0.029	3725	25,781	0.0000
K21	Gastro-oesophageal reflux disease	0.619	0.023	3497	26,009	0.0000
K22	Other diseases of oesophagus	0.621	0.023	2619	26,887	0.0000
R10	Abdominal and pelvic pain	0.672	0.033	1419	28,087	0.0000
K25	Gastric ulcer	0.613	0.022	772	28,734	0.0000
**CXX**	**Malignant neoplasm of oesophagus (C15) or stomach (C16)**	**0.761**	**0.048**	**763**	**28,743**	**0.0000**
**R13**	**Dysphagia**	**0.719**	**0.011**	**762**	**28,744**	**0.0000**
K26	Duodenal ulcer	0.694	0.039	626	28,880	0.0000
**C15**	**Malignant neoplasm of oesophagus**	**0.773**	**0.051**	**536**	**28,970**	**0.0000**
**B37**	**Candidiasis**	**0.703**	**0.126**	**469**	**29,037**	**0.0000**
K31	Other diseases of stomach and duodenum	0.612	0.059	401	29,105	0.0001
I85	Oesophageal varices	0.650	0.003	332	29,174	0.0000
R12	Heartburn	0.587	0.107	312	29,194	0.0420
R11	Nausea and vomiting	0.648	0.068	279	29,227	0.0000
C16	Malignant neoplasm of stomach	0.693	0.066	245	29,261	0.0000
K92	Other diseases of digestive system	0.697	0.030	222	29,284	0.0000
D13	Benign neoplasm of other and ill-defined parts of digestive system	0.593	0.142	210	29,296	0.0948
D37	Neoplasm of uncertain or unknown behaviour of oral cavity and digestive organs	0.595	0.092	136	29,370	0.0728
D48	Neoplasm of uncertain or unknown behaviour of other and unspecified sites	0.512	0.131	127	29,379	0.5610
**K91**	**Postprocedural disorders of digestive system, not elsewhere classified**	**0.753**	**0.215**	**120**	**29,386**	**0.0000**
K57	Diverticular disease of intestine	0.543	0.196	93	29,413	0.4163
K20	Oesophagitis	0.640	0.066	86	29,420	0.0136

All targets which reached an area under the receiver operating curve (AUC, mean ± standard deviation [std]) of above 0.70 are highlighted in bold. N pos./neg. exams = number of positive/negative examinations (with and without diagnosis, respectively). *P-val.* P-value for examination scores between groups.

**Table 2:** Target diseases and classifier performance for the colonoscopy dataset.

ICD	Diagnosis	AUROC mean	AUROC 95% CI	N pos exams	N neg. exams	p-val
K57	Diverticular disease of intestine	0.691	0.020	3403	15,538	0.0000
D12	Benign neoplasm of colon, rectum, anus and anal canal	0.694	0.013	3192	15,749	0.0000
K64	Haemorrhoids and perianal venous thrombosis	0.613	0.033	1657	17,284	0.0000
**R10**	**Abdominal and pelvic pain**	**0.707**	**0.013**	**800**	**18,141**	**0.0000**
**CXX**	**Malignant neoplasm of colon (C18) or rectum (C20)**	**0.733**	**0.005**	**631**	**18,310**	**0.0000**
**K50**	**Crohn disease [regional enteritis]**	**0.729**	**0.053**	**514**	**18,427**	**0.0000**
**K51**	**Ulcerative colitis**	**0.775**	**0.068**	**447**	**18,494**	**0.0000**
C18	Malignant neoplasm of colon	0.686	0.051	332	18,609	0.0000
**C20**	**Malignant neoplasm of rectum**	**0.749**	**0.045**	**309**	**18,632**	**0.0000**
A09	Other gastroenteritis and colitis of infectious and unspecified origin	0.639	0.063	247	18,694	0.0001
K92	Other diseases of digestive system	0.646	0.073	239	18,702	0.0000
**I84**	**Haemorrhoids**	**0.930**	**0.029**	**189**	**18,752**	**0.0000**
K60	Fissure and fistula of anal and rectal regions	0.593	0.028	171	18,770	0.0233
**K91**	**Postprocedural disorders of digestive system, not elsewhere classified**	**0.899**	**0.041**	**153**	**18,788**	**0.0000**
K55	Vascular disorders of intestine	0.620	0.067	151	18,790	0.0085
K62	Other diseases of anus and rectum	0.574	0.163	130	18,811	0.2370
R19	Other symptoms and signs involving the digestive system and abdomen	0.551	0.107	114	18,827	0.3459
**T88**	**Other complications of surgical and medical care, not elsewhere classified**	**0.740**	**0.226**	**82**	**18,859**	**0.0000**
L30	Other dermatitis	0.542	0.152	62	18,879	0.4652

All targets which reached an area under the receiver operating curve (AUC, mean ± standard deviation [std]) of above 0.70 are highlighted in bold. N pos./neg. exams = number of positive/negative examinations (with and without diagnosis, respectively). *P-val.* P-value for examination scores between groups.

### Deep learning

For each diagnosis code, we trained a deep convolutional neural network (ResNet-18) with a transfer learning workflow implemented in Python with Pytorch and Fastai. We used three-fold cross-validation in the primary patient cohort, splitting the list of examinations into three partitions, training a network on two partitions, and testing on the third partition. This was repeated three times so that each examination was part of the test set exactly once. All images from the same examination were always in the same cross-validation fold. Subsequently, all statistics were averaged based on the results in each test partition. This workflow ensured that images from the same examination were never part of the training set and test set in the same experiment. We trained each diagnosis for a maximum of 40 epochs and stopped training once validation loss stops decreasing, by using 20% of all images in each training run as an internal validation set. Fine tuning method from Fastai was used during training, which first trains the newly added deep layers of the pretrained ResNet-18 (pretrained on ImageNet) while the shallow layers are freezed and subsequently trains all layers unfreezing all layers of the model with a discriminative learning rate, meaning that the learning rate gradually decreases towards the shallow layers of the model. We used a mini batch size of 128, an initial learning rate of 2E-03. Usually, patients had 5–10 images available, all of which were included in the analysis. For all patients who had more than 20 images, only 20 randomly selected images were used to avoid their over-representation in the data set. Because the distribution of ground truth labels among the examinations was highly imbalanced, we randomly undersampled the more abundant class until class balance was reached on an image level. For the test set, no such undersampling was performed. For deployment of trained models, a numerical (image-level soft prediction) and categorical (image-level hard prediction) output was generated. The image-level hard predictions were aggregated on the level of examinations and the fraction of positively predicted images was used as an examination-level prediction score. This workflow followed a previously validated workflow which was used for weakly-supervised classification of histology images^25^. For external validation experiments in the categories B37, C15 and C16 (gastroscopy) and C18, C20 and K57 (colonoscopy), we trained a convolutional neural network on all images in the primary cohort (Aachen) and evaluated the classification performance on a separate validation set (Wuerzburg) by using the same procedures and hyperparameters as for the within-cohort experiments. All experiments were run on a computing server in the clinical computing infrastructure. The server ran Windows Server 2019 and was equipped with four NVIDIA RTX6000 GPUs.

### Statistics

The primary statistical endpoint was the receiver operating curve (ROC) and the area under the ROC (AUROC) obtained by comparing the examination-level prediction scores to the ground truth label for each examination. 95% confidence intervals were calculated on the distribution of AUROCs for the three folds. Furthermore, examination-level prediction scores were compared with an unpaired, two-tailed t-test between examinations with “present” and “absent” (true and false) ground truth labels for each diagnosis category and the p-value was reported. P-values were considered significant if they were below 0.05. No formal correction for multiple testing was performed.

### Explainability

To ensure explainability of the deep learning system, two approaches were used. First, for the highest scoring images were extracted from the test sets. Highest scoring images were defined as the 2 images with the highest image-level prediction score from the 10 examinations with the highest examination-level prediction score. Second, on these images, Gradient-weighted Class Activation Mapping (Grad-CAM) was applied to generate region-specific importance maps highlighting relevant image regions. Grad-CAM can utilize the gradients of any layer belonging to a certain class to visualize heatmaps. In this study, we computed the gradients of the last convolutional layer for each diagnosis and used their mean value within the feature map along with activations to generate the Grad-CAM mappings.

### Reader study

To improve explainability and identify potential biases in the system, we performed a systematic reader study, i.e. a manual review of the 60 highest scoring images (2 highest scoring images for each of the 30 highest scoring examinations)^28^ for each of the 23 disease categories in the gastroscopy data set. A trained observer with experience in endoscopy (> 600 gastroscopy, > 300 colonoscopy examinations) categorized each of these 60 × 23 = 1380 images into one of five categories: (1) device (e.g. the endoscope [in retroflexion], forceps, clips, stent or other objects were visible on the image, or narrow band imaging was used), (2) artifact (e.g. out of focus images), (3) typical morphology (i.e. an image which showed a disease that was conceivably linked to the ground truth diagnosis label). All other images were classified as (4) non-typical morphology (i.e. images which did not fall into categories 1–3 and which either showed a normal, non-pathological scenery or an unrelated disease). In addition, a category “missing image” was used in examinations which only had a single image. These categories assigned by the reader were used to determine the plausibility of predictions obtained by weakly supervised training.

## Results

### Weakly supervised AI can predict diseases from raw gastroscopy images

**Figure 1:**
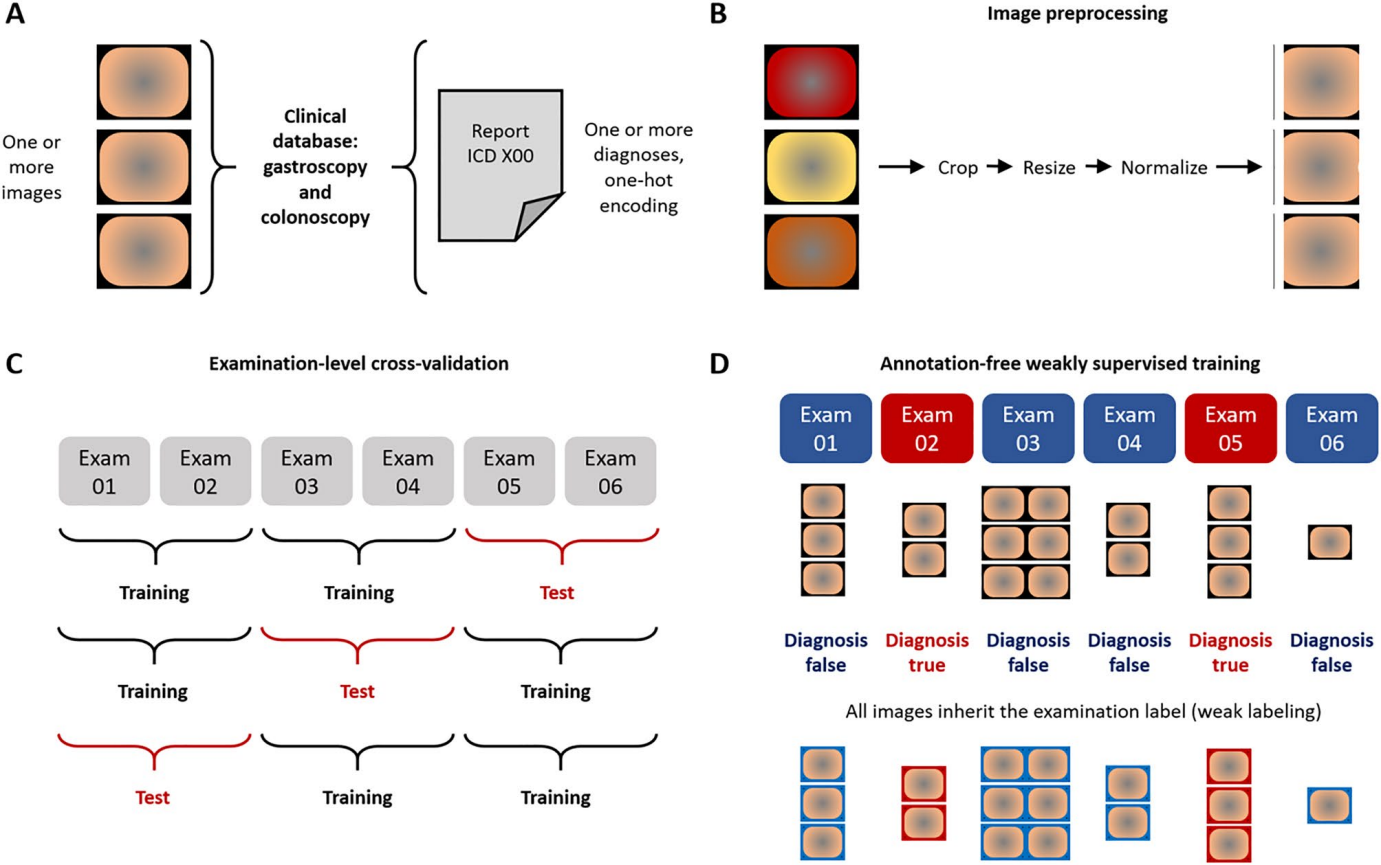
Outline of this study. (**A**) From a large clinical database of gastroscopy and colonoscopy examination, we retrieved images (left) and matched them to disease diagnoses obtained from the corresponding reports (right). (**B**) Images were slightly preprocessed by cropping, resizing and color-normalization by adaptive histogram equalization. (**C**) The experimental workflow relied on examination-level three-fold cross-validation, in which three artificial intelligence (AI) networks were trained for each disease category and the test set was rotated. (**D**) In the weakly supervised AI workflow, all images from a given examination (Exam) inherited the one-hot-encoded disease label. No image-level or pixel-level annotations were used.

**Figure 2:**
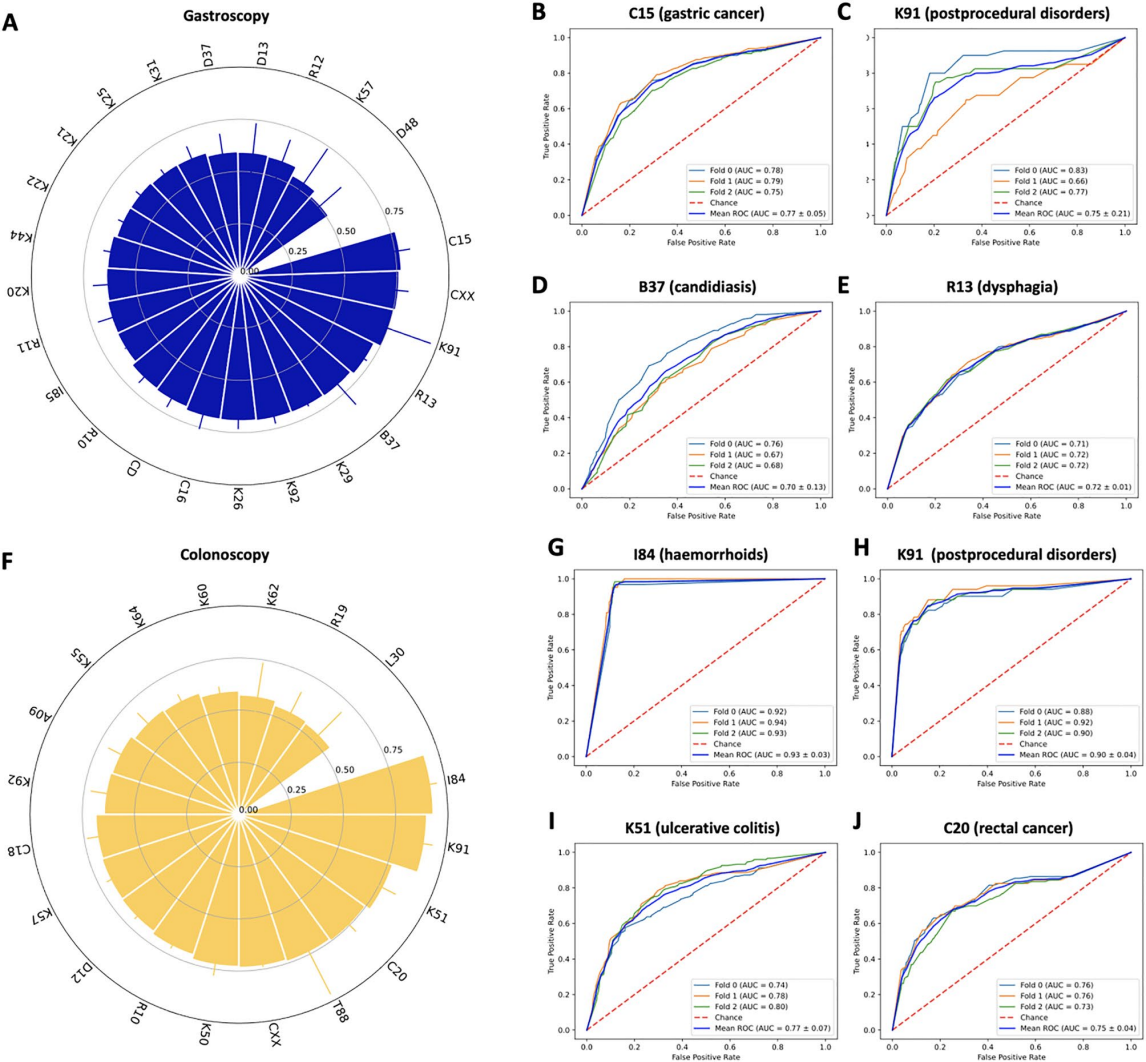
Classification performance for end-to-end artificial intelligence in the gastroscopy and colonoscopy data set. (**A**) Classifier performance in the gastroscopy data set as measured as the mean area under the receiver operating curve (AUROC) of three cross-validation experiments for each disease category with 95% confidence intervals. (**B**) Receiver operating curve (ROC) for C15, (**C**) for K91, (**D**) for B37 and (**E**) for R13. (**F)** AUROCs for all disease categories in the colonoscopy data set and ROCs for (**G**) disease category I84, (**H**) for K91, (**I**) for K51 and (**J**) for C20.

In this study, we used weakly supervised AI to diagnose 43 diseases in 373,174 routine images of gastrointestinal endoscopy (Fig. 1A–D), using the AUROC as the main statistical endpoint. We found that prediction of diagnosis in gastroscopy images in patients in the test set yielded high AUROCs (> 0.70) for five diseases (Fig. 2A). The AUROC was 0.773 (95% confidence interval [CI] 0.051) for C15 (Malignant neoplasm of oesophagus), 0.761 (CI 0.048) for CXX (C15 or C16, malignancy of esophagus or stomach, Fig. 2B), 0.753 (95% CI 0.215) for K91 (Postprocedural disorders, Fig. 2C) and 0.703 (CI 0.126) for B37 (esophageal candidiasis, i.e., thrush, Fig. 2D). In addition to these clearly defined disorders with known morphological correlates, a high AUROC of 0.719 (CI 0.011) was reached for R13 (Dysphagia, Fig. 2E), which is associated with a wide range of esophageal and gastric disorders (Table 1). Five common diagnoses had more than 1000 positive patients per diagnosis class in the gastroscopy dataset: K29 (Gastritis and duodenitis), K44 (Diaphragmatic hernia), K21 (Gastro-oesophageal reflux disease), K22 (Other diseases of oesophagus) and R10 (Abdominal and pelvic pain, Table 1). AI-based detection of these diagnosis classes in image data was achieved with AUROCs between 0.672 (for R10) and 0.698 (for K29, p-value < 0.0001 for all, Table 1).

### Weakly supervised AI can predict diseases from raw colonoscopy images

Similarly, in the colonoscopy dataset, we trained the weakly supervised AI system to detect common diagnoses directly from non-annotated image data. We found that 8 out of 19 disease categories reached AUROCs above 0.70 and 2 of them reached AUROCs close to 0.90 (Fig. 2F, Table 2). The best performance value was achieved for I84 (Haemorrhoids) with an AUROC of 0.930 (CI 0.029, Fig. 2G). However, the disease category I84 for Hemorrhoids only included 189 patients in the dataset because, while the broader category K64 (Haemorrhoids and perianal venous thrombosis) included 1657 patients. K64 achieved an AUROC of 0.613 (CI 0.033, Table 2). The second-best performance was achieved for K91 (Postprocedural disorders) with an AUROC of 0.899 (CI 0.041, Fig. 2H). Similarly to K91, T88 (Other complications of surgical and medical care) reached an AUROC of 0.740 (CI 0.226). Notable diseases with a known association to specific morphological patterns which reached high prediction performance values included K51 (Ulcerative colitis) with an AUROC of 0.775 (CI 0.068, Fig. 2I) and C20 (Malignant neoplasm of rectum) with an AUROC of 0.749 (CI 0.045, Fig. 2J). Other notable disease categories which achieved high performance values included K50 (Crohn disease) with an AUROC of 0.729 (CI 0.053), malignant neoplasm of colon or rectum (CXX, a combination of C18 and C20) with an AUROC of 0.733 (0.005) and the unspecific diagnosis R10 (Abdominal and pelvic pain) which reached an AUROC of 0.707 (CI 0.013).

### The AI system identifies plausible images and potential confounders

**Figure 3:**
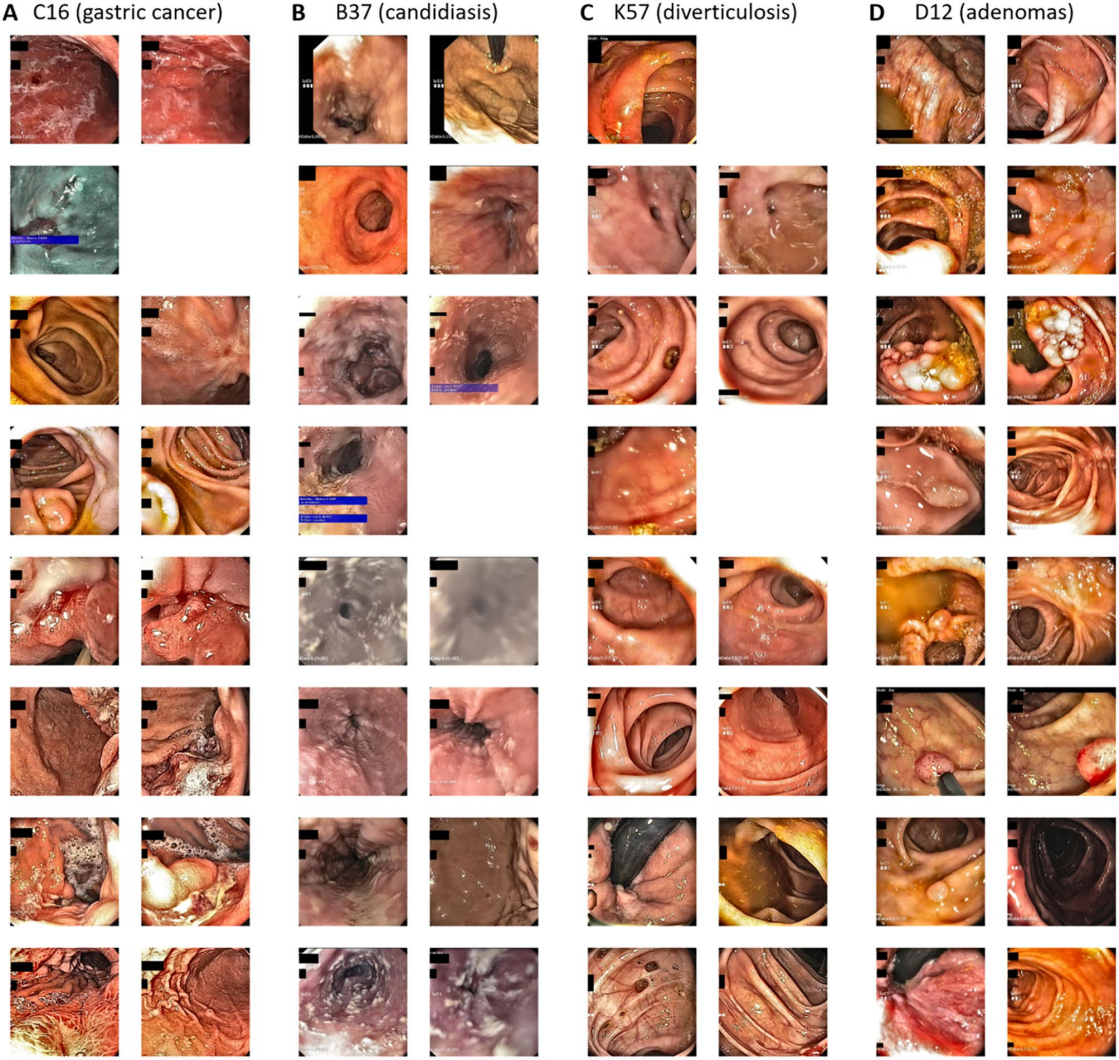
Highly scoring images identified by the artificial intelligence system in the gastroscopy and colonoscopy data set. The two highest scoring images for the eight highest scoring patients are shown for the gastroscopy dataset: (**A**) C16, (**B**) B37, and the colonoscopy data set: (**C**) K57 and (**D**) D12.

**Figure 4:**
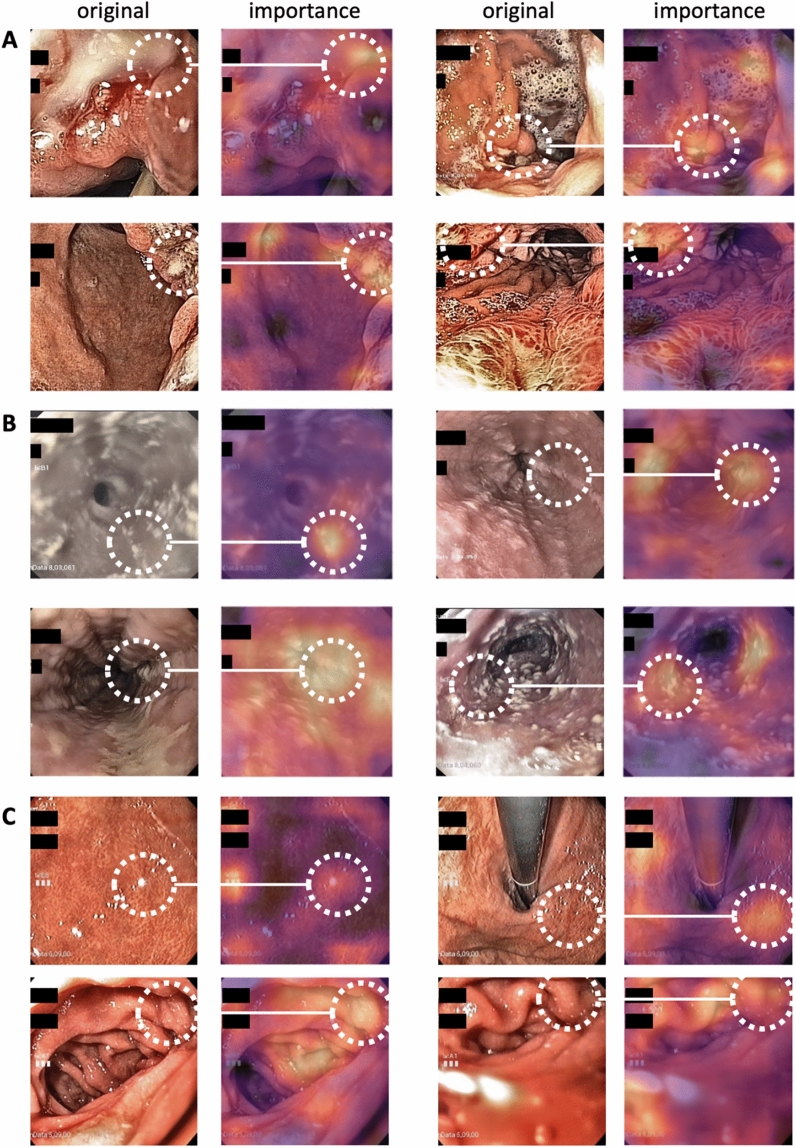
Importance maps for highly predictive image regions in the gastroscopy dataset. (**A**) Representative images with corresponding importance maps (generated by GradCAM) for C16 (gastric cancer). (**B**) Representative images for B37 (candidiasis). (**C**) Representative images for K29 (Gastritis and duodenitis). White circles indicate corresponding areas in the original image and the GradCAM heatmap.

Like any AI systems, weakly supervised approaches can be influenced by biases in the source data. To investigate if high classification performance for disease categories was related to the detection of plausible patterns in images, we developed and used two approaches: identification of highly predictive images and identification of highly predictive regions within these images. First, we extracted and analyzed the two highest scoring images for the ten highest scoring patients for four representative disease categories: C16 (gastric cancer, Fig. 3A), B37 (candidiasis, Fig. 3B), K57 (diverticulosis, Fig. 3C) and D12 (adenomas, Fig. 3D). We found that in all of these categories, despite the high degree of label noise in the original dataset, highly typical morphological patterns were present. For example, a range of lesions which are highly suspect for malignancy were present in the gastric cancer (C16, Fig. 3A) highly predictive images, including diffusely inflammatory patterns and localized polypoid lesions. Also, for B37 (esophageal candidiasis), the highest scoring images (Fig. 3B) almost exclusively showed highly typical morphological patterns, despite the fact that C16 and B37 had AUROCs of around 0.70 (Table 2). Next, we investigated if these images were selected by the AI system because of the presence of plausible patterns in these images. We used GradCAM to generate “importance maps” on the highest predictive images and manually reviewed the maps (representative maps shown in Fig. 4A,B). Indeed, we found that for all diagnosis categories, plausible image regions were highlighted by GradCAM, for example polypoid tumors for gastric cancer (C16, Fig. 4A) and white lesions corresponding to thrush esophagitis for B37 (candidiasis, Fig. 4B). However, this analysis also identified potential confounders: in the top left image of Fig. 4C, an artifact (light reflection) in the gastric mucosa was erroneously assigned a high importance score for K29 (gastritis and duodenitis). Finally, we quantified the ability of the AI system to link plausible morphology to disease categories by systematically reviewing the highest scoring 60 images (20 images from each of the three folds) for each disease category in the gastroscopy dataset and classifying images as having a typical (or plausible) morphology, no typical morphology or missingness, presence of endoscopic devices as potential confounders or image artifacts (Table 3). In this reader study, we found that for B37 (Candidiasis), K26 (Duodenal ulcer), K20 (Oesophagitis), K22 (Other diseases of oesophagus) and C15 (Malignant neoplasm of oesophagus), more than 50% of the highly scoring images were highly typical or plausible for the respective disease, which means that the AI system successfully identified images associated with diseases from a large non-annotated database. For a number of ICD diagnoses, a substantial proportion (> 20%) of highly predictive images showed medical devices (acting as potential biases, Suppl. Fig. S1A–I), including retroverted endoscopes, stents and clips, in particular CXX (C15 or C16, malignant neoplasm of esophagus or stomach, 28% of images with devices, Table 3), I85 (esophageal varices, 20%), K91 (postprocedural disorders, 42%) and K44 (diaphragmatic hernia, 30%). Together, these data show that without any image- or pixel-level annotation, an AI system can identify plausible visual patterns linked to disease, but manual review can identify some potential biases.

**Table 3:** Results of the reader study in the gastroscopy data set.

ICD	Diagnosis	Typical	Not typical	Missing	Device	Artifact
B37	Candidiasis	0.68	0.18	0.03	0.07	0.03
K26	Duodenal ulcer	0.60	0.23	0.00	0.15	0.02
K20	Oesophagitis	0.57	0.32	0.02	0.08	0.02
K22	Other diseases of oesophagus	0.55	0.15	0.22	0.05	0.03
C15	Malignant neoplasm of oesophagus	0.50	0.13	0.13	0.18	0.05
K29	Gastritis and duodenitis	0.48	0.23	0.03	0.18	0.07
C16	Malignant neoplasm of stomach	0.48	0.33	0.05	0.13	0.00
K25	Gastric ulcer	0.47	0.33	0.10	0.08	0.02
K92	Other diseases of digestive system	0.47	0.35	0.03	0.15	0.00
D13	Benign neoplasm of other and ill-defined parts of digestive system	0.42	0.32	0.07	0.18	0.02
K21	Gastro-oesophageal reflux disease	0.42	0.38	0.02	0.18	0.00
CXX	Malignant neoplasm of oesophagus or stomach	0.40	0.17	0.12	0.28	0.03
R11	Nausea and vomiting	0.38	0.37	0.08	0.13	0.03
K31	Other diseases of stomach and duodenum	0.37	0.35	0.02	0.22	0.05
D37	Neoplasm of uncertain/unknown behaviour, oral cavity/digestive organs	0.37	0.43	0.02	0.17	0.02
R13	Dysphagia	0.28	0.47	0.12	0.10	0.03
I85	Oesophageal varices	0.27	0.48	0.03	0.20	0.02
D48	Neoplasm of uncertain/unknown behaviour of other/unspecified sites	0.25	0.43	0.07	0.23	0.02
K57	Diverticular disease of intestine	0.20	0.68	0.00	0.10	0.02
R12	Heartburn	0.18	0.65	0.05	0.10	0.02
K91	Postprocedural disorders of digestive system, not elsewhere classified	0.17	0.28	0.10	0.42	0.03
K44	Diaphragmatic hernia	0.12	0.57	0.00	0.30	0.02
R10	Abdominal and pelvic pain	0.10	0.75	0.08	0.05	0.02

For each diagnosis category in the gastroscopy dataset, the AI system selected 60 highly predictive images (2 highest scoring images from 10 highest scoring patients in each of the 3 folds). These images were manually classified into five categories by a trained observer. The fraction of images in each category in every category is shown.

### Generalizability to an external validation set

**Figure 5:**
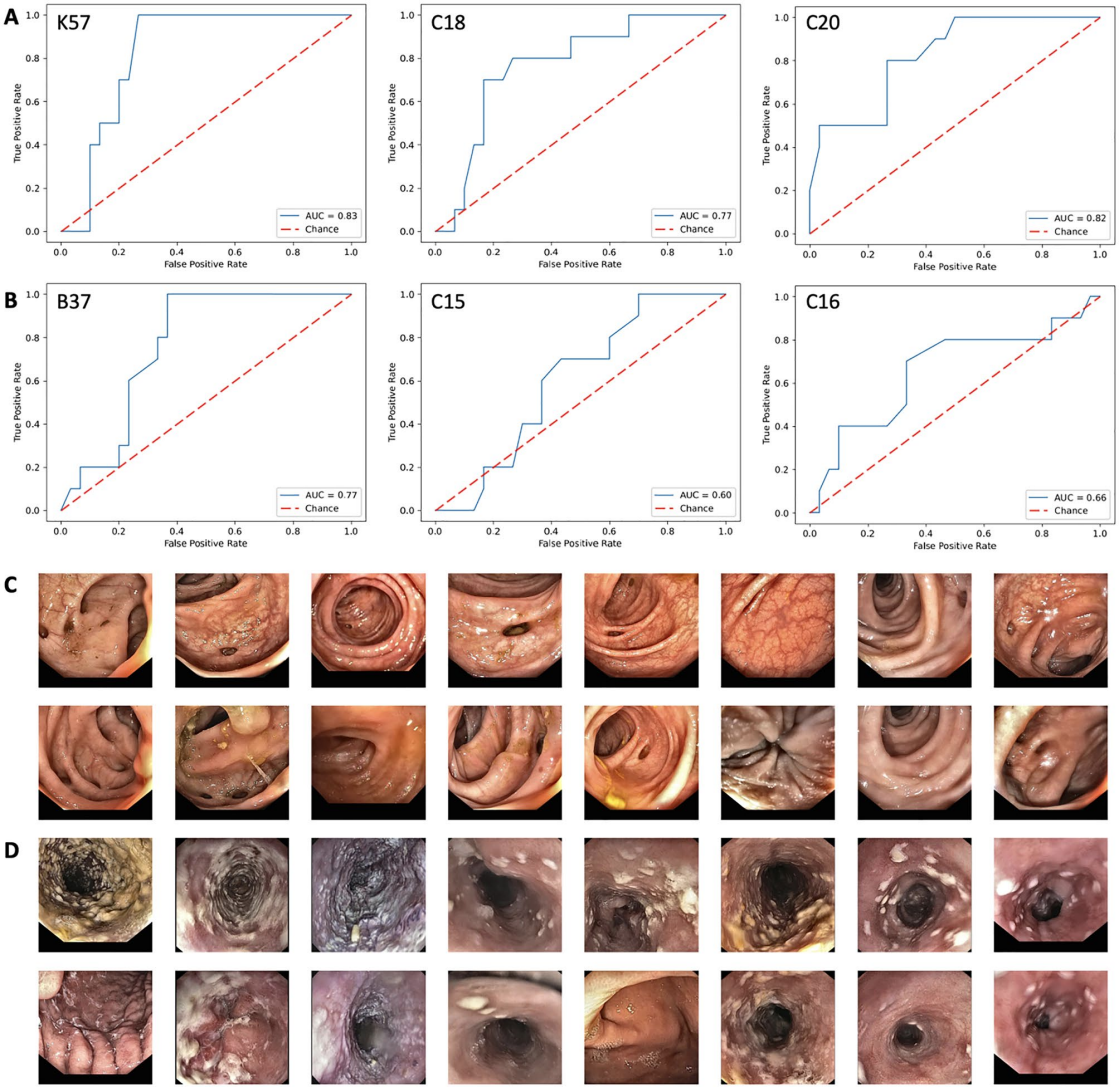
Classification performance in the external validation set. (**A**) Receiver operating curves (ROC) with area under the curve (AUC) in the colonoscopy validation set for K57 (diverticulosis), C18 (colon cancer) and C20 (rectal cancer). (**B**) ROC in the gastroscopy validation set for B37 (candidiasis), C15 (esophageal cancer) and C16 (gastric cancer). (**C**) Highest scoring images (two each) in highest scoring patients (eight) as selected by the AI model from the validation set for K57 (diverticulosis) (**D**) and B37 (candidiasis).

AI classifiers are prone to overfit to the training dataset, in particular in weakly supervised settings in which latent biases might skew performance. Therefore, we externally validated the performance of our proposed AI system on six diagnosis categories in gastroscopy and colonoscopy examinations from a separate validation cohort. In the colonoscopy data set, this approach reached AUROCs of 0.832 (p = 0.0044), 0.772 (p = 0.0182) and 0.825 (p = 0.0004) for K57 (diverticular disease), C18 (colon cancer) and C20 (rectal cancer), respectively (Fig. 5A, Suppl. Table S1), outperforming the within-cohort classifiers (Table 2). In the gastroscopy data set, this approach reached AUROCs of 0.767 (p = 0.0097), 0.603 (p = 0.2989) and 0.667 (p = 0.145), for B37 (candidiasis), C15 (esophageal cancer) and C16 (gastric cancer), respectively (Fig. 5B). In addition, a manual review of the highly scoring images as detected by the AI system in the validation set revealed the presence of highly plausible patterns: for K57 (diverticulosis), all of the eight highest scoring patients in the validation set showed the presence of a diverticulum (Fig. 5C). Similarly, all images selected by the AI system for B37 (candidiasis) showed the typical presence of white esophageal lesions (Fig. 5D). Together, these results demonstrate that the pathognomonic patterns learned by the AI system in the weakly labeled training set were robustly generalizable to an external validation cohort.

## Discussion

### Weakly supervised AI creates new opportunities for endoscopy image analysis

Here, we show that AI can detect some of the most common diseases in routine images of upper and lower gastrointestinal (GI) endoscopy, including malignant, infectious and inflammatory diseases. Compared to previous studies of AI in endoscopy which relied on laborious manual annotation of images, our approach relies only on examination-level labels extracted from routine endoscopy reports. For training, we use a non-annotated routine database from a high-volume endoscopic center, including a multitude of endoscopic devices, spanning multiple years. Neither the image data nor the report data were specifically intended for analysis by an AI system at the time of the examination. Yet, the weakly supervised AI system achieves high predictive performance for identification of diagnoses in endoscopy image data (Fig. 2). By making predictions explainable on an image and sub-image scale, we show that the AI system learns highly plausible patterns at the level of images (Fig. 3) and regions (Fig. 4). We show that the resulting classifiers are robust and generalize well to a separate validation cohort, in which the classification performance in the original dataset is even surpassed for several diagnostic categories (Fig. 5). Together, these findings are a strong proof of concept for the usefulness of weakly supervised AI systems in clinical routine image data even in the absence of any expert-based annotations.

Our study extends the use of AI in gastroenterology from curated and highly selected datasets to the vast amount of routine image data stored on clinical servers in endoscopy departments across the world. AI systems in general, and weakly supervised systems in particular, are potentially susceptible to biases in the distribution of ground truth labels^29^. One of the most important prerequisites for medical AI is that it provides explainability to gain trust among users and allow identification of biases^30^. While our weakly supervised AI system achieved high diagnostic performance and enables explainability, it is not immune to biases in the image dataset. In this study, we identify potential confounders in our approach but also demonstrate that clinically relevant patterns are correctly identified by our approach. In summary, we show for the first time that AI can learn visual patterns of upper and lower GI pathology directly from examination labels in a weakly supervised way.

### Limitations

The main limitations of our study are related to the fact that all images in this study were originally not obtained with quantitative image analysis in mind, but were intended for human interpretation and documentation. This resulted in a large variation in terms of image quality, the number of images per case and the anatomic regions represented in these images. In this study, we compensate for these variations by using hundreds of thousands of images. Another way to address these problems in the future is by routinely recording any endoscopic examination, thereby limiting the bias introduced by human operators who choose when to save an image to the archive. From a technical point of view, the structure of the current dataset can lead to label noise and potential biases in the dataset which this study aimed to identify and quantify. Label noise limits this study because all images in a given exam inherit the exam label, which we know is not correct. For example, a patient with a diaphragmatic hernia will still have images of the normal duodenum in their file. Again, due to the high number of cases, we empirically show that AI can compensate for this type of label noise and learn clinically plausible image patterns. Another type of label noise is due to the imperfect nature of the diagnoses which were made during the endoscopy procedure and not by histopathology, which should be the ultimate gold standard for future validation studies. Our approach enabled us to also address unspecific diagnoses such as R12 (heartburn) or R13 (dysphagia), which were among the most common diagnoses in the dataset. Unlike diagnoses with a clear morphological correlation (such as K20, oesophagitis), it is unclear if prediction of such an unspecific diagnosis is useful. Yet, in our study we demonstrate that even for diagnostic categories like R12 (heartburn) plausible image features are learned by the AI system, including reflux esophagitis. Based on this finding we hypothesize that AI systems could even detect disease states which are not (yet) obvious to the endoscopist, hence the unspecific symptom-based diagnosis in the endoscopy report. Future studies are needed to validate this hypothesis. Another potential limitation of our primary dataset is the presence of text information on some images, which might in theory have been exploited by the trained models. We addressed this problem by evaluating classifiers on a separate patient cohort without any text on the images. Yet, for future studies, endoscopy images should be saved without any text overlay. In addition, our analysis was done on the level of examinations, not on the level of patients and future studies. Finally, our weakly supervised approach enabled us to analyze a large, non-annotated dataset but the performance could be inferior to a fully supervised approach. Future studies need to weigh the advantages and disadvantages of such weakly supervised versus fully supervised approaches.

### Outlook

In this study, we report that weakly supervised AI systems can achieve a high performance and maintain explainability in end-to-end image analysis in GI endoscopy. This shows that manual annotations are not necessarily a bottleneck for future clinical applications of AI. Thus, weakly supervised AI could enable the analysis of large, multi-centric databases of millions of endoscopy examinations without being limited by manual annotation. In addition, the capacity of AI systems to learn pathophysiologically relevant patterns from non-annotated images shows that AI could enable mechanistic scientific insight in addition to yielding tools of potential clinical usefulness. Future approaches could also be used to predict clinical features as well as genomic findings and predict risk of malignancy in conjunction with other data elements such as clinical, radiology, and whole slide images^31^. Our study provides a blueprint as well as the algorithms and open-source implementation for future multicentric studies required to implement weakly supervised AI systems as diagnostic support tools in endoscopy.

## Supplementary Information

Supplementary Information.

## Data Availability

The images used in this study contain patient-related data (names, date of birth) and this study was approved by the institutional review board under the provision that all analyses are carried out within the clinical computing servers of University Hospital Aachen. Therefore, the image data cannot be made available. If external parties wish to use the dataset for validation of other AI models, they can contact the study principal investigators (LB and JNK) who will seek approval by the institutional review board within four weeks.

## Code availability

All source codes for the deep learning training and deployment are publicly available under an open-source license at https://github.com/KatherLab/deepmed with an extensive user manual^28^.
