# Supplementary material for: Weakly supervised end-to-end artificial intelligence in gastrointestinal endoscopy
Source: Sci Rep. 2022 Mar 22;12:4829. doi: 10.1038/s41598-022-08773-1 (PMC8941159; doi:10.1038/s41598-022-08773-1)
Supplement: Supplementary file 1 — Supplementary Information. [file 41598_2022_8773_MOESM1_ESM.pdf]

Supplementary Figures and Tables

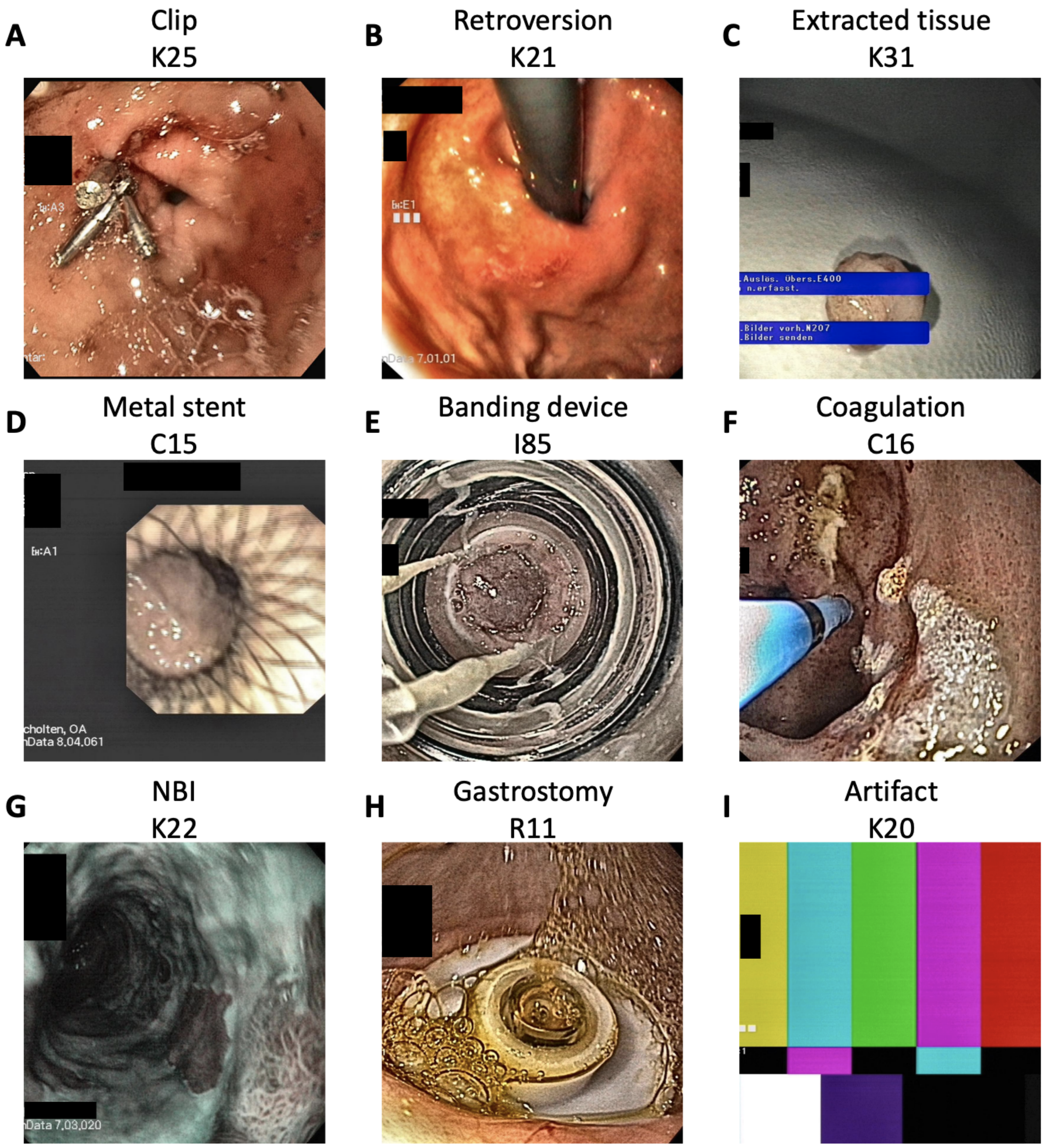

Suppl. Figure S1: Potential confounders in the gastroscopy dataset. Images which were classified as "devices" or "artifacts" in the reader study of highly predictive images. The corresponding disease category which these images were part of is shown above each panel. Common devices which were identified included **(A)** metal clips, **(B)** retroverted endoscopes, **(C)** extracted tissue or objects such as polyps after polypectomy, **(C)** metal stents, **(E)** endoscopic banding devices and **(F)** argon plasma coagulation probes. **(G)** In addition, the presence of narrow band imaging in images was classified as the use of a device with potential confounding effect. **(G)** Gastrostomy tubes and **(I)** image artifacts were also present in a small fraction of highly predictive images.

ICD	Diagnosis	AUROC	AUROC 95% CI	N pos. exams	N neg. exams	p-val.
K57	Diverticular disease of intestine	0.832	N/A	10	30	0.0044
C18	Malignant neoplasm of colon	0.772	N/A	10	30	0.0182
C20	Malignant neoplasm of rectum	0.825	N/A	10	30	0.0004
B37	Candidiasis	0.767	N/A	10	30	0.0097
C15	Malignant neoplasm of oesophagus	0.603	N/A	10	30	0.2989
C16	Malignant neoplasm of stomach	0.667	N/A	10	30	0.145

Suppl. Table S1: Classifier performance in the external validation set. No confidence intervals for AUROCs are given because only a single inference run was performed. The categories in the validation set were selected to have 3 colonoscopy and 3 gastroscopy diagnoses, of which 1 was non-neoplastic and 2 were neoplastic.
